# Advisory Committee on Immunization Practices Recommended Immunization Schedules for Persons Aged 0 Through 18 Years — United States, 2015

**Published:** 2015-02-06

**Authors:** Raymond A. Strikas, Advisory Committee on Immunization Practices (ACIP), ACIP Child/Adolescent Immunization Work Group

**Affiliations:** 1 Immunization Services Division, National Center for Immunization and Respiratory Diseases, CDC

Each year, the Advisory Committee on Immunization Practices (ACIP) reviews the recommended immunization schedules for persons aged 0 through 18 years to ensure that the schedules reflect current recommendations for Food and Drug Administration–licensed vaccines. In October 2014, ACIP approved the recommended immunization schedules for persons aged 0 through 18 years for 2015, which include several changes from the 2014 immunization schedules. For 2015, the figures, footnotes, and tables are being published on the CDC immunization schedule website (http://www.cdc.gov/vaccines/schedules/index.html). This provides readers electronic access to the most current version of the schedules and footnotes on the CDC website. Health care providers are advised to use figures, tables, and the combined footnotes together. Printable versions of the 2015 immunization schedules for persons aged 0 through 18 years also are available at the website in several formats, including portrait, landscape, and pocket-sized versions. Ordering instructions for laminated versions and “parent-friendly” schedules also are available at the immunization schedule website.

For further guidance on use of each vaccine included in the schedules, including contraindications and precautions when using a vaccine, health care providers are referred to the respective ACIP vaccine recommendations at http://www.cdc.gov/vaccines/hcp/acip-recs. In addition, changes in recommendations for specific vaccines can occur between annual updates to the childhood/adolescent immunization schedules.

*Recommendations for routine use of vaccines in children, adolescents, and adults are developed by the Advisory Committee on Immunization Practices (ACIP). ACIP is chartered as a federal advisory committee to provide expert external advice and guidance to the Director of the Centers for Disease Control and Prevention (CDC) on use of vaccines and related agents for the control of vaccine-preventable diseases in the civilian population of the United States. Recommendations for routine use of vaccines in children and adolescents are harmonized to the greatest extent possible with recommendations made by the American Academy of Pediatrics (AAP), the American Academy of Family Physicians (AAFP), and the American College of Obstetrics and Gynecology (ACOG). Recommendations for routine use of vaccines in adults are harmonized with recommendations of AAFP, ACOG, the American College of Physicians (ACP), and the American College of Nurse Midwives (ACNM). ACIP recommendations adopted by the CDC Director become agency guidelines on the date published in the* Morbidity and Mortality Weekly Report (MMWR). *Additional information regarding ACIP is available at http://www.cdc.gov/vaccines/acip.*

These immunization schedules are approved by ACIP (http://www.cdc.gov/vaccines/acip/index.html), the American Academy of Pediatrics (http://www.aap.org), the American Academy of Family Physicians (http://www.aafp.org), and the American College of Obstetricians and Gynecologists (http://www.acog.org).

The most current immunization schedules can be found on the Vaccines and Immunizations pages of CDC’s website (http://www.cdc.gov/vaccines/schedules). If errors or omissions are discovered, CDC posts revised versions on these web pages. CDC encourages organizations that previously have relied on copying the schedules on their websites to instead use syndication to consistently display schedules that are current. This is a more reliable and accurate method and ensures that the most current and accurate immunization schedules are on each organization’s website.

Use of content syndication requires a one-time step that ensures that an organization’s website displays current schedules as soon as they are published or revised. Instructions for the syndication code are available at http://www.cdc.gov/vaccines/schedules/syndicate.html. CDC offers technical assistance for implementing this form of content syndication. Assistance from a website staff member is available via e-mail at ncirdwebteam@cdc.gov .

Changes to the previous schedules† include the following:

- Figure 1, “Recommended Immunization Schedule for Persons Aged 0 through 18 Years” was modified to highlight the recommendations for influenza vaccination for children 1) for live attenuated influenza vaccine, which may only be administered beginning at age 2 years, and 2) for children aged 6 months through 8 years, who need 2 doses of influenza vaccine in the first year vaccinated, and in subsequent years only require 1 dose of vaccine. Therefore, the gold bar for live attenuated influenza vaccine (LAIV) or inactivated influenza vaccine (IIV) 1 or 2 doses extends from 2 through 8 years (midpoint of column for 7–10 years) and a new gold bar (1 dose) extends from 9 to 18 years to reflect these changes.
- A purple bar was added for measles-mumps-rubella (MMR) vaccine for children aged 6–11 months, denoting the recommendation to vaccinate such children if they will travel or live abroad.
- Pages 4 through 6 contain combined footnotes for each vaccine related to routine vaccination, catch-up vaccination,§ and vaccination of persons with high-risk medical conditions or special circumstances.
- Standardized formatting is used for footnotes for each vaccine to reflect the number of vaccine doses in a particular series.
- The diphtheria/tetanus/acellular pertussis (DTaP) vaccine footnote has language added stating if the fourth dose DTaP vaccine was administered 4 months or more after the third dose, at an appropriate age, it can be counted as a valid dose, and need not be repeated after the recommended 6-month interval between doses 3 and 4.
- The meningococcal conjugate vaccine footnote was revised to more clearly present recommendations for use of MenACWY-CRM, MenACWY-D, and Hib-MenCY-TT in children aged 2 months and older with anatomic or functional asplenia, or with persistent complement deficiencies.
- The influenza vaccine footnote was updated to reflect revised contraindications for LAIV: LAIV should not be administered to some persons, including 1) persons who have experienced severe allergic reactions to LAIV, any of its components, or to a previous dose of any other influenza vaccine; 2) children aged 2 through 17 years receiving aspirin or aspirin-containing products; 3) persons who are allergic to eggs; 4) pregnant women; 5) immunosuppressed persons; 6) children aged 2 through 4 years with asthma or who had wheezing in the past 12 months; and 7) persons who have taken influenza antiviral medications in the previous 48 hours. All other contraindications and precautions to use of LAIV are available at http://www.cdc.gov/mmwr/pdf/wk/mm6332.pdf.
- The pneumococcal vaccine footnote was updated to provide clearer guidance for vaccination of persons with high-risk conditions:
  – Administer 1 dose of PCV13 if any incomplete schedule of 3 doses of PCV (PCV7 and/or PCV13) was received previously.
  – Administer 2 doses of PCV13 at least 8 weeks apart if unvaccinated or any incomplete schedule of fewer than 3 doses of PCV (PCV7 and/or PCV13) was received previously.
- Figure 2, Catch-Up Immunization Schedule: *Haemophilus influenzae* type b (Hib) conjugate vaccine, pneumococcal conjugate vaccine, and tetanus, diphtheria, acellular pertussis (Tdap), and varicella vaccine catch-up schedules were updated to provide more clarity. Minimum ages were noted as “not-applicable” for children aged 7 years and older for hepatitis A and B, polio, meningococcal, MMR, and varicella vaccines.

In addition to the updated schedule figures and footnotes, CDC has developed “job-aids” with detailed scenarios by age group and previous doses of vaccine received for DTaP, Hib, and pneumococcal conjugate vaccines. These materials should assist health care providers in interpreting Figure 2, the Childhood/Adolescent Immunization catch-up schedule. The job-aids are available at http://www.cdc.gov/vaccines/schedules/hcp/child-adolescent.html.

## Footnotes

† Past immunization schedules are available at http://www.cdc.gov/vaccines/schedules/past.html.

§ For persons aged 4 months through 18 years who start late or who are more than 1 month behind in receiving recommended vaccinations.

